# White Matter Evaluation in Multiple Sclerosis Through Magnetic Resonance Kurtosis Imaging

**DOI:** 10.7759/cureus.6424

**Published:** 2019-12-19

**Authors:** Sevim Sahin, Isa Çam, Onural Öztürk, Husnu Efendi, Yonca Anık, Ozcan Gundogdu

**Affiliations:** 1 Biomedical Engineering, Kocaeli University, Kocaeli, TUR; 2 Radiology, Faculty of Medicine, Kocaeli University, Kocaeli, TUR; 3 Neurology, Faculty of Medicine, Kocaeli University, Kocaeli, TUR

**Keywords:** multiple sclerosis, diffusion kurtosis imaging, mri

## Abstract

Objectives: To investigate diffusional changes in multiple sclerosis (MS) plaques and non-Gaussian behavior of water diffusion by using diffusional kurtosis imaging (DKI).

Methods: 31 MS patients and 21 controls underwent MRI on a 3T scanner. Mean kurtosis (MK) parametric maps were computed. Region of interest (ROI) was delineated as white matter (WM) in controls and MS plaques and WM in patients.

Results: There was no significance of WM kurtosis and skewness parameters among MS group and control group patients p=0.213 and p=0.390, respectively. In MS patients, kurtosis, skewness, maximum intensity, minimum intensity, and median intensity values of WM, Plaque 1, Plaque 2, and Plague 3 were significantly higher at p<0.0001 for all.

Conclusions: DKI may provide more extensive characterization of lesions and WM and may be a sensitive indicator of tissue damage and microstructural change in patients with MS in addition to conventional diffusional evaluations.

## Introduction

Multiple sclerosis (MS) is a chronic inflammatory demyelination disease of central nervous system. It may cause a prominent level of neurological disability in young adults and is identified by the formation of focal demyelinated plaques in the WM [1]. The myelin damage is the key component of MS pathogenesis. Focal plaques or the lesions are the primary focus for imaging myelin in MS, which appear bright on proton density and T2 weighted imaging [2]. In studies, myelin water fraction (MWF) is decreased differentially in MS lesions [3-4]. However, diffusion analysis of water molecules using MRI is a quantitative technique to obtain more specific microstructural information than conventional MRI.

 Diffusion kurtosis imaging (DKI) is an imaging technique, which provides further information of non-Gaussian water diffusion behaviors, particularly in neural tissue, based on diffusion properties of water molecules [5]. DKI is an expansion of diffusion tensor imaging (DTI) where diffusion tensor is estimated together with a fourth order 3D kurtosis tensor. In theory, in the Gaussian diffusion model, the water molecules diffuse uniformly in a certain direction. In DTI the diffusion-weighted signal resembles a monoexponential decay as shown in Eq. (1).

$(ln\left \lfloor \frac{S(b)}{S_{}0} \right \rfloor= -bD_{app})$ (1)

However, human body has complicated cellular structures and the water molecules diffuse through a media, which is excessively nonhomogeneous in all directions, conducive to deviation from the Gaussian distribution. Excess kurtosis term (*K*) is included in the DKI to catch this deviation from the Gaussian distribution shown in Eq. (2) [6].

$(ln\left \lfloor \frac{S(b)}{S_{}0} \right \rfloor= -bD_{app}+\frac{1}{6}b^{2}D_{app}^{2}K_{app})$ (2) 

*D*_app_: the apparent diffusion coefficient

*K*_app_: the kurtosis along a certain diffusion direction

*S*_(*b*)_: the diffusion-weighted signal along that direction with a certain *b* value

*S*_0_: the nondiffusion-weighted signal

The mean kurtosis (MK) corresponding to the diffusional kurtosis (DK) averaged over all gradient directions is shown in Eq. (3) [7].

$(\bar{K}\equiv \frac{1}{4\pi }\int d\Omega _{n}K\left ( n \right ))$ (3)

dΩ_n_ : solid angle element for direction *n.*

The standard DTI metrics, such as apparent diffusion coefficient (ADC) and fractional anisotropy (FA) can be obtained by DKI. DK is not based on spatially oriented tissue structures and it allows us to evaluate both gray matter (GM) and WM simultaneously which is an advantage. Furthermore, crossing fiber tracts does not affect DKI differently from DTI [8]. 

Recently, studies have shown that DKI is useful to evaluate the microstructural environment of brain tissue [9-15]. In this study, we aim to evaluate diffusional changes on MS plaques by using DKI.

## Materials and methods

Institutional review board of Kocaeli University approved this study. Written informed consent was obtained from all participants.

Subjects

A total of 52 consecutive patients (32 female and 20 male) participated in this study. Some 31 patients diagnosed with MS according to McDonald 2010 criteria [16] and 21 healthy controls with no known brain abnormalities and no neurological symptoms were included.

MRI acquisition

 Imaging was performed by using a 3T MR scanner (Achieva; Philips Medical Systems, Netherlands) with 16-channel head phased array coil. After routine contrasted cranial MRI sequences, including T1 and T2 weighted images and fluid-attenuated inversion recovery (FLAIR) images, axial diffusion tensor image data were acquired. The parameters used for DTI were as follows; repetition time (ms)/ echo time (ms) 10077/55, section thickness/gap 2/0 mm; 60 sections, field of view (FOV) 224 x 224, matrix 112 x 112, voxel size 0.88 x 0.88 x 2 mm^3^, and five *b* values (0, 500, 1000, 1500, and 2000 s/mm^2^) with diffusion encoding in 16 directions in every *b* value.

Image postprocessing

Images were transferred to an offline workstation. All DICOM images were converted to 4D NIFTI by using the free software dcm2niix, which is a subpart of MRIcroGL [NeuroImaging Tools & Resources Collaboratory (NITRC)], then converted to a series of 3D NIFTI files by using dcm2niigui (by NITRC). As 500, 1000, and 1500 isoweighted images were repeated due to Philips’ acquisition protocol, these images were removed. Following, 3D NIFTI images, excluding the isoweighted images were restacked by using dcm2niigui.

Diffusion kurtosis parametric maps were calculated by using the free software diffusion kurtosis estimator (DKE), Version 2.6; in Matlab, Version 7.0 [17]. MK, FA, and mean diffusivity (MD) parametric maps were obtained. The DKI fitting method was chosen as directional linear weighted to improve image quality.

Region of interest analysis

**Figure 1:**
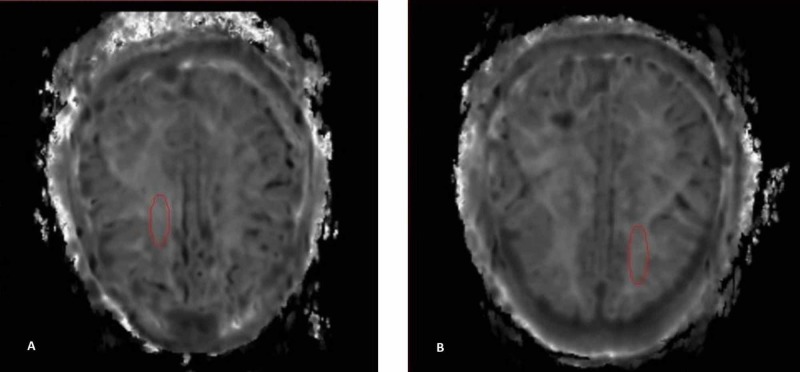
White matter images of a) control group patient and b) MS patient

**Figure 2:**
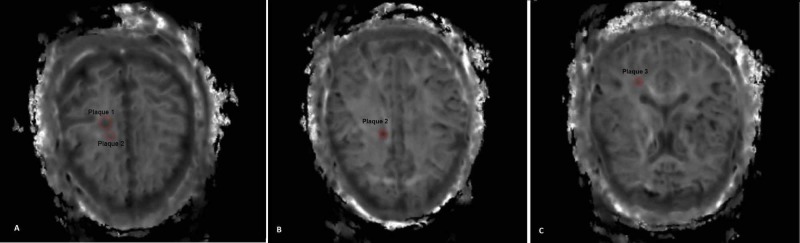
MS lesions at different slices for different patients:  A) Plaque 1 and Plaque 2 B) Plaque 2 and C) Plaque 3. MS, multiple sclerosis

All MK maps and T2 FLAIR images were fused and controlled by a radiologist and a medical physicist, individually. The MS lesions and WM were outlined on T2 weighted images by using the free software Medical Image Processing, Analysis, and Visualizations (MIPAV) developed by the National Institutes of Health Center for Information Technology (CIT). Figure 1A shows MK images of WM for a control group and Figure 1B shows MK images of an MS patient. The MS plaques were delineated according to their size and brightness of T2 FLAIR images. Three largest MS plaques were assessed for each patient. The lesions with the highest size and brightness were determined as Plaque 1. Plaque 2 and Plaque 3 lesions were outlined according to decreasing size and brightness, respectively (please see Figure 2A-C). The delineated regions of interest (ROI) were automatically transferred onto the corresponding MK maps and the kurtosis, skewness, minimum intensity, maximum intensity, and median intensity values were measured. Skewness values are generated automatically via kurtosis values.

Statistical analysis

Statistical evaluation was performed using SPSS 13.0. Age, sex, mean value, and standard deviation of MS and the control group were performed by simple definition test. While WM is evaluated in the control group, plaques and WM are evaluated in the MS group. T test was performed to assess the significant differences in the values of skewness, kurtosis, maximum intensity, minimum intensity, and median intensity in MS group and control group and oneway ANOVA post hoc test was performed on Plaque 1, Plaque 2, Plaque 3, and WM in MS patients.

## Results

Demographic data of subjects

There was no significant difference between MS and control groups in relation to sex (p=0.604). The median age of MS group and the control group was 39.32 ± 9.904 years and 38.29 ± 10.926 years, respectively. No significant difference was seen between groups in relation to median age (p=0.721). Age and sex characteristics of MS and control groups are summarized in Tables 1 and 2, respectively.

**Table 1:** Age characteristics of subjects.

	N	Minimum	Maximum	Median	Std deviation
Multiple sclerosis age	31	24	56	39.32	9.904
Control age	21	22	60	38.29	10.626

**Table 2:** Gender characteristics of subjects.

		Frequency	Ratio
Multiple sclerosis	Female	20	64,5
	Male	11	35,5
	Total	31	100,0
Control	Female	12	57,1
	Male	9	42,9
	Total	21	100,0

DKI values of MS and control group

**Figure 3:**
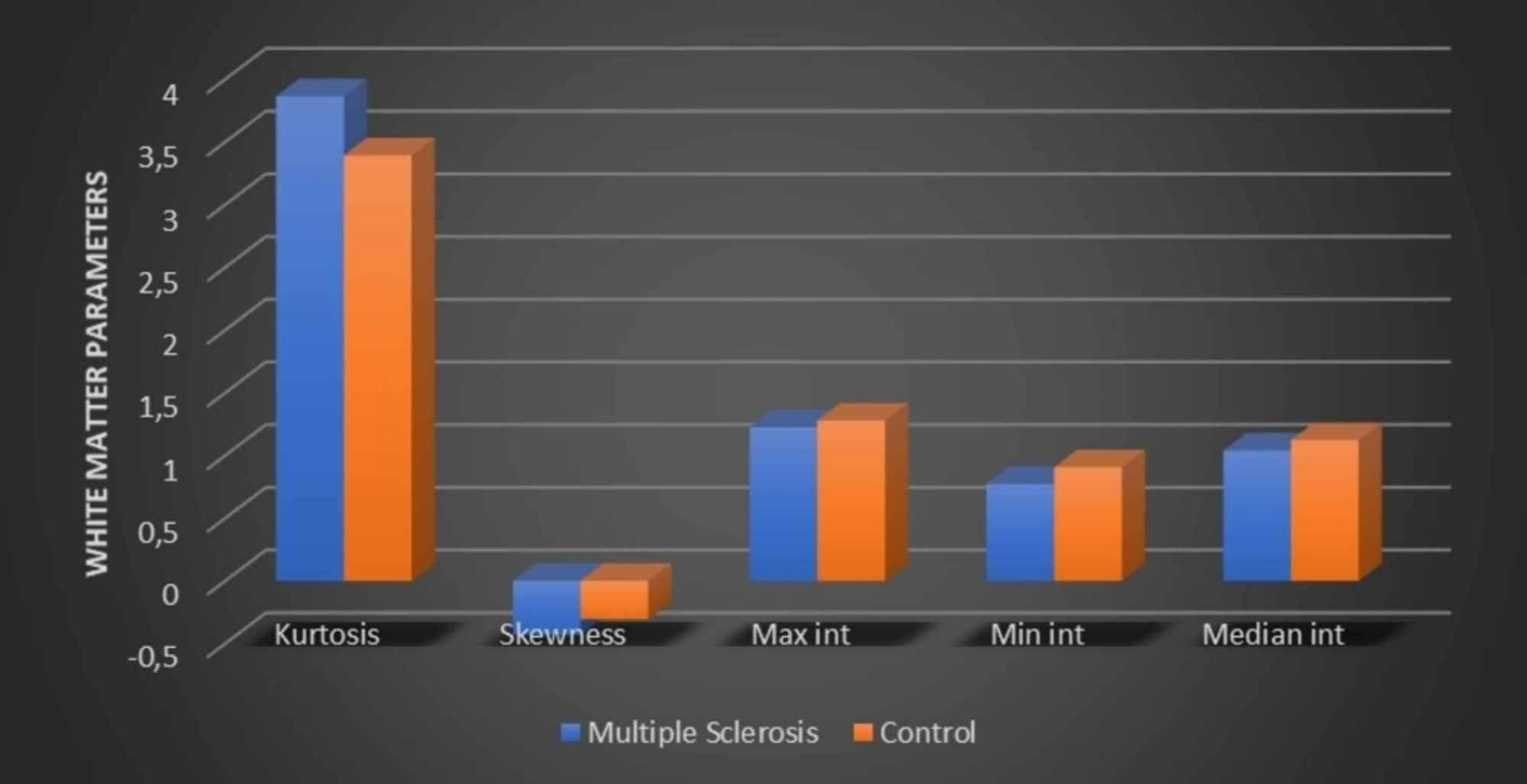
White matter characteristics between MS and control groups. MS, multiple sclerosis

There was no significance of WM kurtosis and skewness parameters among MS group and control group patients p=0.213 and p=0.390, respectively. These analyses are shown in Figure 3.

**Table 3:** MS group results. MS, multiple sclerosis

MS group					
	Mean				
	Kurtosis	Skewness	Maximum intensity	Minimum intensity	Median intensity
White matter	3.672969	-0.380333	1.245475	0.826392	1.071567
Plaque 1	2.638493	0.181370	1.151790	0.515163	0.791913
Plaque 2	2.507323	0.028663	1.087263	0.512243	0.792313
Plaque 3	2.463060	0.054363	1.123723	0.607573	0.853230
Total	2.952540	-0.083418	1.166535	0.648041	0.907361

In MS patients, kurtosis, skewness, maximum intensity, minimum intensity, and median intensity values of WM, Plaque 1, Plaque 2, and Plaque 3 were significantly higher at p<0.0001 for all patients. Maximum intensity, minimum intensity, and median intensity were significantly lower in MS patients compared with that of control group patients p=0.034, p=0.001, p=0.004 respectively. MS group results are summarized in Table 3.

**Figure 4:**
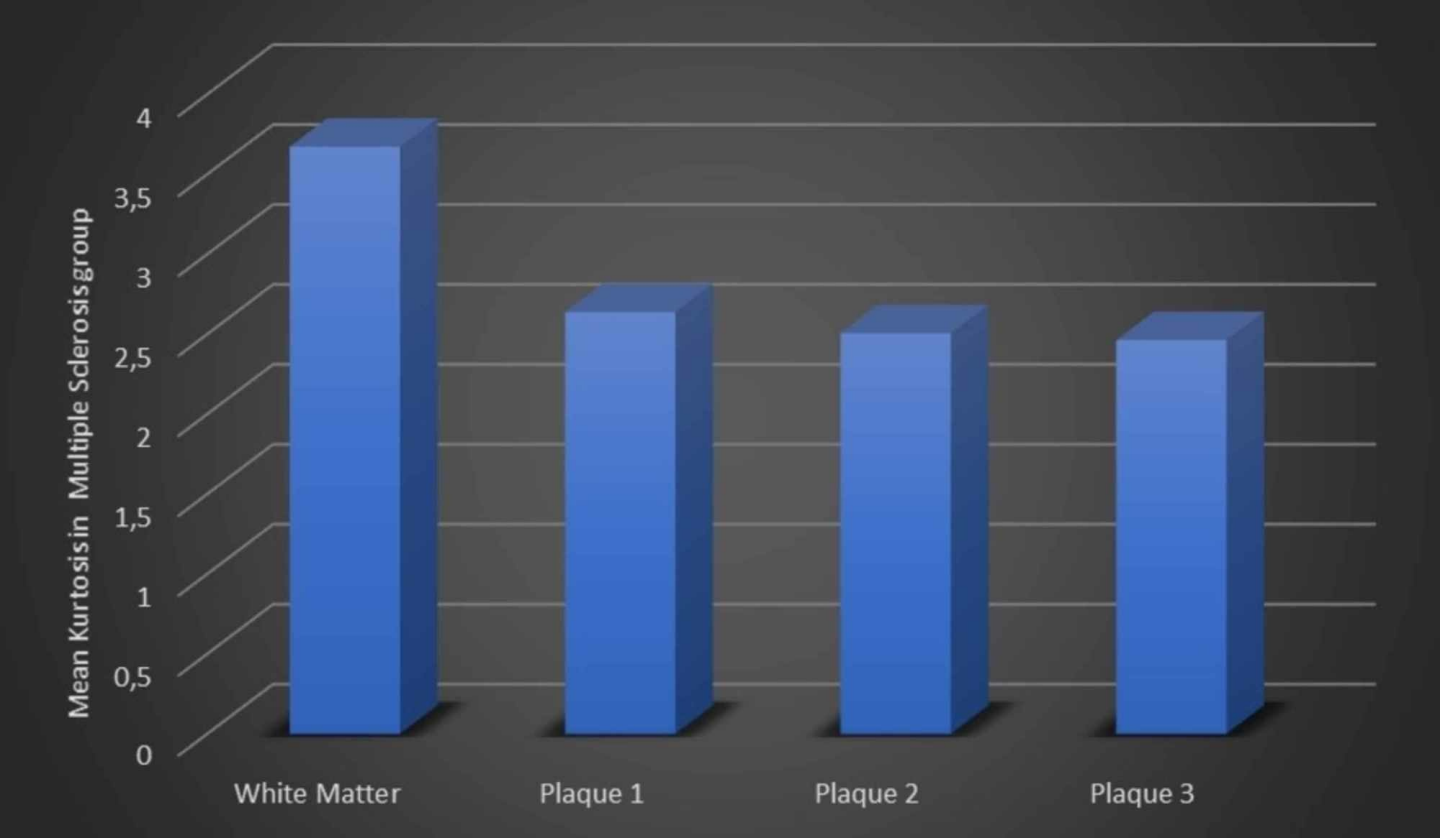
MK of white matter, Plaque 1, Plaque 2, and Plaque 3 of MS group. MK, mean kurtosis; MS, multiple sclerosis

Figure 4 shows that MK values of WM are significantly higher than the MS plaques.

## Discussion

The diffusion of water in the brain is considered as Gaussian distribution in DTI, thereby the diffusional heterogeneity cannot be determined in biological tissues as the diffusion in brain is restricted by water compartments, barriers of cell membranes, myelin layers, organelles axon sheaths, etc. DKI which is a mathematical extension of DTI is able to quantify the non-Gaussian water diffusion behavior by providing additional and different diffusional metrics. Diffusion kurtosis does not base upon spatially oriented tissue structures that make it more advantageous than FA. Thus, DKI can be used as an indicator of microstructural complexity and used to examine abnormalities in tissues with isotropic structure such as GM and WM [18]. 

Our results showed that the biggest and brightest plaques give more significant differences. In MS group, MK of WM, Plaque 1, Plaque 2, and Plaque 3 were significantly higher at p<0.0001 which can be explained by that the decrease of kurtosis could be a sign of neuronal loss [19]. Unless there was no significance observed between WM in the control group and normal appearing white matter (NAWM) in MS group.

Yoshida et al. [20] showed that DKI was able to detect abnormalities in NAWM that could not be imaged by conventional MRI. In their study, a significant difference (p=0.002) between WM and NAWM on MK has been shown between six controls and 11 MS patients. In another study, Zhang et al. [21] showed that relative to normal tissue, MS plaques have increased diffusivity [axial (*D*_ax_), radial (*D*_rad_), and MD] but decreased kurtosis [axial (*K*_ax_), radial (*K*_rad_), and MK].

Limitations

 Diffusional kurtosis imaging is very sensitive to the effects of signal noise due to use of higher *b* values. In our study, we used five *b* values (0, 500, 1000, 1500, and 2000 s/mm^2^) and we had low resolution images due to high noise and low signal to noise ratio (SNR). The combination of *b* values can also be important to obtain proper DKI data due to the noise sensitivity and in future studies *b* value combinations for particular disease might be investigated. Furthermore, noise correction would be required for strong diffusion weightings.

## Conclusions

Diffusional kurtosis imaging is a clinically feasible method that has the potential to provide new information on the brain tissue microstructure that is provided by conventional diffusion weighted imaging and DTI metrics. Future diffusion kurtosis imaging studies might include combination of neurological tests for MS lesion locations. Thus, disease progression at an early stage might be monitored through the use of DKI. More studies are needed to determine disease burden and disability.

## References

[1] Lasmann H, Brück W, Lucchinetti CF (2007). The immunopatholgy of multiple sclerosis: an overview. Brain Pathol.

[2] MacKay AL, Laule C (2016). Magnetic resonance of myelin water: an in vivo marker for myelin. Brain Plasticity.

[3] Laule C, Vavasour IM, Moore GRW (2004). Water content and myelin water fraction in multiple sclerosis: a T2 relaxation study. J Neurol.

[4] Oh J, Han ET, Lee MC (2007). Multi-slice brain myelin water fractions at 3T in multiple sclerosis. J Neuroimaging.

[5] Wu EX, Cheung MM (2010). MR diffusion kurtosis imaging for neural tissue characterization. NMR Biomed.

[6] Jensen JH, Helpern JA, Ramani A (2005). Diffusional kurtosis imaging: the quantification of non-gaussian water diffusion by means of magnetic resonance imaging. Magn Reson Med.

[7] Jensen JH, Helpern JA (2010). MRI quantification of non-Gaussian water diffusion by kurtosis analysis. NMR Biomed.

[8] Hori M, Fukunaga I, Masutani Y (2012). Visualizing non-gaussian diffusion: clinical application of q-space imaging and diffusional kurtosis imaging of the brain and spine. Magn Reson Med.

[9] Kouchkovsky I, Fieremans E, Fleyser L (2016). Quantification of normal-appearing white matter tract integrity in multiple sclerosis: a diffusion kurtosis imaging study. J Neurol.

[10] Zhuo J, Xu S, Proctor JL (2012). Diffusion kurtosis as an in vivo imaging marker for reactive astrogliosis in traumatic brain injury. Neuroimage.

[11] Grossman EJ, Ge Y, Jensen JH (2012). Thalamus and cognitive impairment in mild traumatic brain injury: a diffusional kurtosis imaging study. J Neurotrauma.

[12] Falangola MF, Jensen JH, Tabesh A (2013). Non-gaussian diffusion MRI assessment of brain microstructure in mild cognitive impairment and Alzheimer’s disease. Magn Reson Imaging.

[13] Van Cauter S, Veraart J, Sijbers J (2012). Gliomas: diffusion kurtosis MR imaging in grading. Radiology.

[14] Wang JJ, Lin WY, Lu CS (2011). Parkinson disease: diagnostic utility of diffusion kurtosis imaging. Radiology.

[15] Gong NJ, Wong CS, Chan CC (2013). Correlations between microstructural alterations and severity of cognitive deficiency in Alzheimer’s disease and mild cognitive impairment: a diffusional kurtosis imaging study. Magn Reson Imaging.

[16] Polman CH, Reingold SC, Banwel B (2011). Diagnostic criteria for multiple sclerosis: 2010 revisions to the McDonald criteria. Ann Neurol.

[17] Tabesh A, Jensen JH, Ardekani BA (2011). Estimation of tensors and tensor-derived measures in diffusional kurtosis imaging. Magn Reson Med.

[18] Jensen JH, Helpern JA, Ramani A (2005). Diffusional kurtosis imaging: the quantification of non-gaussian water diffusion by means of magnetic resonance imaging. Magn Reson Med.

[19] Arab A, Vojna-Pelczar A, Khairnar A (2018). Principles of diffusion kurtosis imaging and its role in early diagnosis of neurodegenerative disorders. Brain Res Bull.

[20] Yoshida M, Hori M, Yokoyama K (2013). Diffusional kurtosis imaging of normal-appearing white matter in multiple sclerosis: preliminary experience. Jpn J Radiol.

[21] Zhang Z, Jensen J, Jaggi H (2011). Diffusional kurtosis imaging in multiple sclerosis. Radiological Society of North America 2011 Scientific Assembly and Annual Meeting, November 26-December 2, Chicago, IL.

